# Mental health burden among healthcare workers in Kintampo North Municipal Hospital: A descriptive analysis of stress, depression, and anxiety based on Job Demands-Resources (JD-R) model

**DOI:** 10.1371/journal.pmen.0000478

**Published:** 2025-12-03

**Authors:** Mohammed Zakaria, Dennis Bardoe, Robert Bagngmen Bio, Denis Dekugmen Yar, Daniel Hayford

**Affiliations:** 1 Department of Public Health Education, Akenten Appiah-Menka University of Skills Training and Entrepreneurial Development, Mampong, Ghana; 2 Department of Integrated Science Education, Akenten Appiah-Menka University of Skills Training and Entrepreneurial Development, Mampong, Ghana; 3 Department of Disease Control and Epidemiology, College of Health and Well-Being, Kintampo, Ghana; National University of Singapore, SINGAPORE

## Abstract

Mental health disorders among healthcare workers remain a growing concern, particularly in under-researched settings. While global evidence has documented the burden of these disorders, there is limited empirical data from Kintampo North Municipality. This study assessed the prevalence and correlates of stress, depression, and anxiety among health workers in Kintampo North Municipal Hospital. A hospital-based cross-sectional study involving 316 healthcare workers was conducted at Kintampo North Municipal Hospital. Standardised tools, including the Perceived Stress Scale (PSS-10), Beck Depression Inventory (BDI-21), and Beck Anxiety Inventory (BAI-21), were used to assess stress, depression, and anxiety, respectively. Descriptive statistics, Pearson’s chi-square tests, and multivariate logistic regression analyses were performed using STATA 17. Variables with p ≤ 0.25 in the bivariate model were included in the multivariate model. Adjusted odds ratios (AORs) with 95% confidence intervals (CIs) were reported at a significance level of p < 0. 05. The prevalence of clinically significant stress, depression, and anxiety was 66.5% (95% CI: 61.20 – 71.70), 63.6% (95% CI: 58.30 – 68.90), and 87.9% (95% CI: 84.40 – 91.60), respectively. Key correlates across all the three mental health disorders included job dissatisfaction, rotational shifts, increased workload, chronic illness, alcohol consumption, extended working hours, limited sleep, male gender, and specific occupational roles such as nurses, allied health personnel, physicians, emergency medical technicians, and dispensary technicians. The high burden of the three mental health disorders among healthcare workers in Kintampo North Municipal Hospital highlights deep systemic and occupational challenges within the health system. While these findings point to the resilience of staff working under resource constraints, they also signal the need for targeted institutional reforms. Expanding access to workplace mental health support, ensuring flexible scheduling, reducing mandatory overtime, and addressing job dissatisfaction and workload inequities could be essential to foster a healthier, more sustainable healthcare workforce in Ghana and similar contexts.

## Introduction

According to the World Health Organisation (WHO), mental health encompasses more than the absence of mental disorders; it includes states of psychological well-being in which individuals realise their abilities, cope with normal life stresses, work productively, and contribute to their communities [1]. Depending on the patient’s perceived condition, healthcare workers provide services to improve or maintain anatomical, physiological, and psychological function or, to a larger extent, obtain information about the health status and prognosis [2]. However, despite their critical contributions, healthcare professionals are increasingly vulnerable to psychological distress, including stress, depression, and anxiety, due to the demanding nature of their work [3].

Stress as a psychological disorder stems from emotional and physical strain caused by attempts to respond to internal and external pressures [4]. As a commonly reported mental health disorder, depression causes a chronic feeling of emptiness, sadness, loss of interest in pleasurable activities, guilt, low self-esteem, sleep disturbance, and difficulties in concentration [5]. Anxiety, on the other hand, is how an individual’s body responds to a perceived threat [6]. These disorders often co-occur and are intensified by factors such as long work hours, heavy patient workloads, inadequate resources, and exposure to trauma. If left unaddressed, it may impair professional performance, personal well-being, quality of care, absenteeism, high turnover rates, and compromise patient safety and organisational efficiency [7].

Globally, evidence suggests that healthcare workers experience disproportionately high levels of stress, depression, and anxiety. Before this study, some studies on mental health disorders among healthcare workers have yielded evidence of inter-and intra-country variations. For instance, A U.S. national survey found that 30% of healthcare workers experienced stress during the pandemic [8]. Similarly, depression among healthcare workers is higher in Africa, with a prevalence rate of 82%, followed by the United States of America and Europe, with rates of 33% and 31%, respectively. The lowest prevalence of depression was recorded in Asia, with a prevalence rate of 19% [9]. Furthermore, a cross-sectional study conducted in Jordan among 422 healthcare workers reported a 30.8% prevalence of anxiety [10].

Although numerous studies have explored mental health outcomes among health workers globally. In Ghana, most of the existing studies are concentrated in tertiary facilities or urban centres, overlooking district hospitals and municipalities that face unique challenges. For example, a study by Opoku Agyemang et al. (2022) on psychiatric nurses in Ghana revealed significant levels of anxiety and depression [3]. Similarly, studies from other African countries, such as Nigeria, Sierra Leone, Ethiopia, and Rwanda, have shown that health workers face mental health challenges exacerbated by systemic inefficiencies and lack of support [11,12]. However, there is a significant gap in the literature on health workers’ mental health within smaller health facilities, particularly in resource-constrained municipalities such as Kintampo North in Ghana. Kintampo North Municipality is noted to be a high-risk zone for road traffic accidents and infectious disease outbreaks [13,14]. These public health dynamics exert extra pressure on health workers to deliver timely and efficient health care to people. Despite these contextual challenges, a key issue that remains unknown is the unavailability of peer-reviewed studies that have examined the prevalence and correlates of stress, depression, and anxiety among health workers in Kintampo North Municipal Hospital. Addressing this knowledge gap is essential for developing targeted interventions that improve healthcare workforce resilience, reduce psychological harm, and ensure sustained healthcare delivery. Hence, a dedicated examination was essential to inform targeted interventions and policies tailored to the needs of the local healthcare workforce. Guided by the Job Demands-Resources (JD-R) Model, this study assessed the prevalence and factors associated with stress, depression, and anxiety among health workers at Kintampo North Municipal Hospital. Specifically, it sought to provide insights into the challenges that may differ from those observed in global or national studies, thereby enhancing the effectiveness of interventions and contributing to long-term improvement in mental health. The findings are expected to inform context-specific occupational health strategies and guide national policies focused on improving the mental well-being of healthcare workers across similar high-demand, resource-constrained settings.

## Materials and methods

### Ethics approval and consent to participate

The protocol of the study was reviewed and approved by the Committee on Human Research, Publication, and Ethics (CHRPE), Kwame Nkrumah University of Science and Technology, School of Medical Sciences, in accordance with the Declaration of Helsinki [assigned approval number: CHRPE/AP/037/24]. Participants signed a written consent form after receiving a thorough explanation before engaging in the study.

### Study design and population

The study was a hospital-based cross-sectional study conducted among 316 health workers at Kintampo North Municipal Hospital from 22^nd^ January to 11^th^ June 2024.

### Study setting

The study was conducted in Kintampo North Municipality of the Bono East Region of Ghana. Geographically, the municipality is strategically located at the centre of Ghana and serves as a transit point between the northern and southern sectors of the country [15]. It lies between latitudes 8º45’N and 7º45’N and Longitudes 1º20’W and 2°1’E [15]. The municipality has a general hospital in Kintampo, from which people can receive health care. There are also four private health facilities, two public rural clinics, two health centres, one outreach office, and 12 functional CHPS compounds across the municipality to provide for the health needs of the inhabitants [16].

### Theoretical foundation of the study

This study was grounded in the Job Demands-Resources (JD-R) Model. This model provides a comprehensive framework for understanding how work-related factors impact employee well-being, particularly in high-stress environments like healthcare [17]. The JD-R model posits that job demands lead to burnout and mental health issues when workers are not equipped with adequate job resources to cope with these demands [17]. This model is particularly relevant in healthcare settings, where job demands are often high, and resources may be scarce. The JD-R model divides the factors influencing mental health into two broad categories. These are job demands and job resources [18]. Job demands refer to the aspects of the job that require sustained physical or mental effort, which could lead to stress and burnout when they are excessive or not matched with sufficient resources [18]. In this study, job demands include predictors such as workload, job dissatisfaction, rotational shift patterns, and long working hours, which are expected to increase the risk of stress, depression, and anxiety among healthcare workers. On the other hand, job resources are the factors that help employees cope with job demands and foster well-being [17]. In this study, sleep duration was considered a personal resource that may buffer the impact of job demands on mental health. Adequate sleep is essential for recovery and emotional resilience. This could, in turn, mitigate the negative effects of work-related stressors. In addition, the JD-R model acknowledges the role of individual and demographic factors in moderating the relationship between job demands/resources and mental health outcomes [17,18]. Factors such as gender, age, marital status, and occupational role could influence how job demands are perceived and how effectively employees could utilise available resources to cope with these demands [19]. In this study, these factors are included as covariates, as they may either exacerbate or buffer the impact of job-related stress on healthcare workers’ mental health.

### Conceptual framework of the study

**Fig 1:**
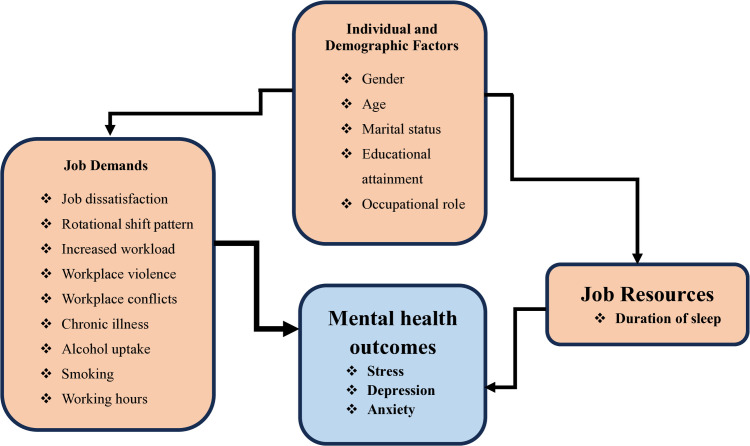
The study’s conceptual framework, informed by the Job Demands-Resources (JD-R) Model.

**Fig 1** illustrates the expected connections between job demands, job resources, individual factors, and mental health outcomes. Conceptually, this study assumes that job demands lead to increased stress, depression, and anxiety. Job resources, on the other hand, act as buffers to mitigate the negative impact of job demands on mental health. Finally, individual and demographic factors may moderate the relationship between job demands or resources and mental health outcomes.

### Inclusion criteria

The study included all health workers at Kintampo North Municipal Hospital who provided consent for participation.

### Exclusion criteria

The study excluded health workers who were not staff of the selected health facility and those who did not provide consent to participate in the study.

### Sample size determination

The sample used in this study was obtained using Slovene’s formula:


$$n=\frac{N}{\text{1}+\text{N}(e{)}^{\text{2}}}$$

Where (**N)** is the target population (437), (**e**) is the standard error (Chosen to be 0.05), and (**n**) is the sample size.


$$=\frac{\text{4}\text{3}\text{7}}{\text{1}+{\text{4}\text{3}\text{7}(\text{0}.\text{0}\text{5})}^{\text{2}}}$$

≈209

After determining the sample size of the 209 health workers, to ensure sufficient statistical power and reliability from the sample for analysis, 55% of the total sample size (209), which is 114.95 rounded to the nearest decimal, was added to give a total sample size of 324. Therefore, the final sample size for the study included 324 health workers. Given the objective of comparing mental health disorders across different units in the hospital, oversampling was employed to prevent the underrepresentation of units with smaller populations. This allowed for equitable distribution and improved the generalizability and precision of subgroup analyses. A post-hoc power analysis was conducted using G*Power (Version 3.1.9.7) to assess whether the sample size of 324 participants is sufficient for detecting significant effects with multiple predictors. The analysis, assuming a medium effect size (f² = 0.15) and 10 predictors, revealed that the study achieves 86% power at a significance level of 0.05. This power value exceeds the standard threshold of 80%, confirming that the sample size is adequate for the planned multivariate logistic regression analysis.

### Sampling procedure

This study employed a multistage sampling technique. Initially, a quota sampling method was used to allocate health workers based on their occupational categories, as shown in **Table 1**. The quotas were initially defined based on the proportion of healthcare workers in each unit of the hospital. After accounting for a 2.46% non-response rate, the quotas were adjusted to maintain proportional representation by distributing non-respondents across the remaining units based on the initial sampling proportions. Within each occupational category, simple random sampling was used to select health workers for participation. Each health worker within a given strata (occupation category) was assigned a unique identification number, and balloting was employed to ensure an equal chance of selection. To prevent underrepresentation from smaller units, oversampling was implemented. After determining a final sample size, an additional 55% of the sample (114.95) was added, bringing the total sample size to 324. This oversampling strategy was to improve precision in subgroup analyses and minimise potential bias in estimating mental health outcomes across various occupational roles [20].

**Table 1:** Proportional allocation of sample size across each occupation category.

Occupation categories	Population	Sample proportion%	Estimated sample size	Approximated sample size
Nurses	338	77.3	250.4	250
Allied Health Personnel	45	10.3	33.4	34
Physicians	23	5.3	17.2	17
Emergency Medical Technicians	14	3.2	10.4	10
Dispensary Technicians	9	2.1	6.8	7
Orderlies	8	1.8	5.8	6
Total	**437**	**100**	**324**	**324**

### Consenting and enrolment of participants

**Fig 2:**
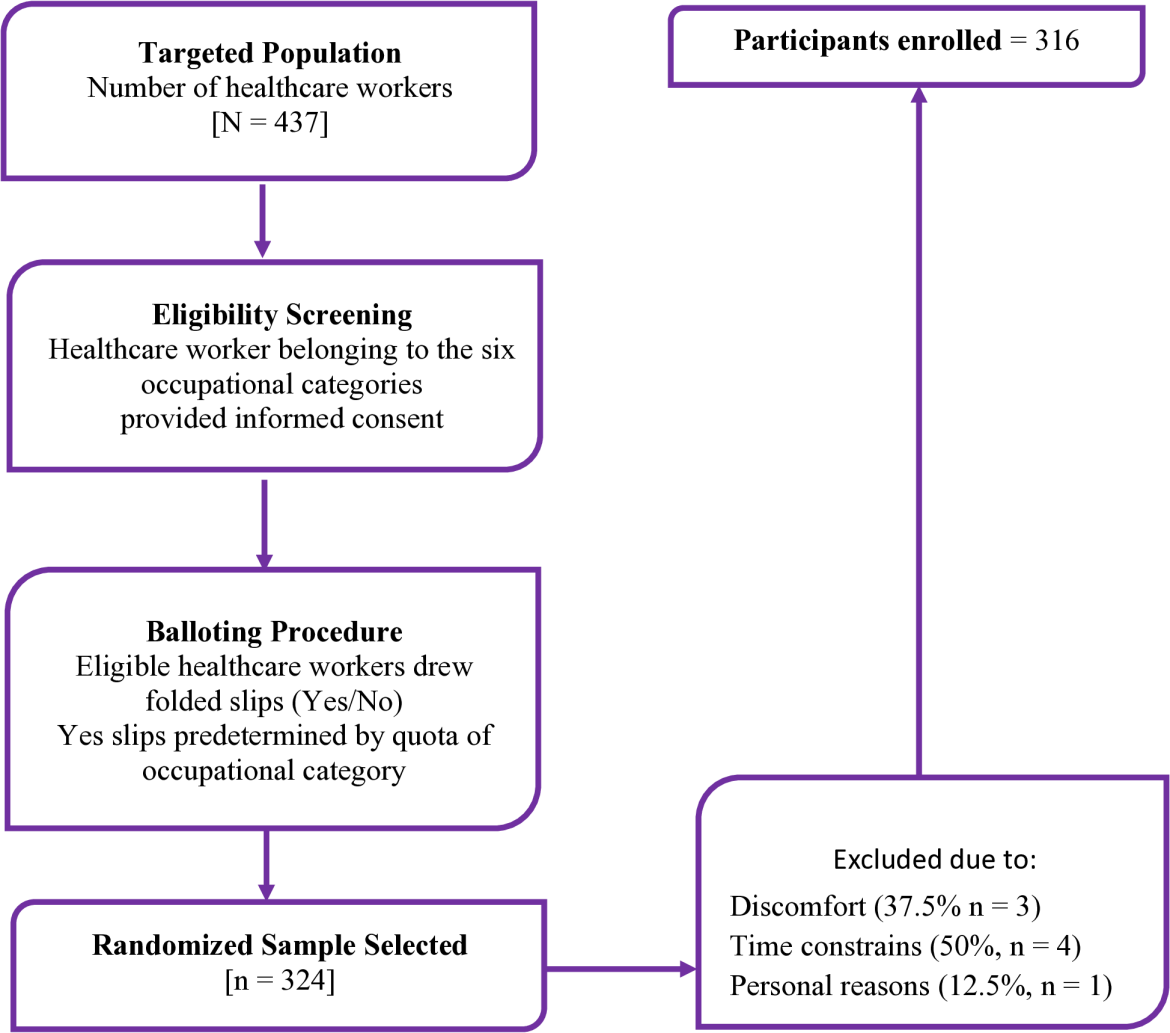
Flowchart of recruitment process.

Health workers were enrolled at various units of Kintampo Municipal Hospital from 22^nd^ January 2024–19^th^ February 2024 (**Fig 2**). After initial screening for eligibility, health workers who met the inclusion criteria were approached to discuss the study and obtain their consent for participation. Consented health workers signed a consent form to affirm their participation.

Variables of interest

Outcome variables

The outcome variables were stress, depression, and anxiety.

### Main predictor variables

The predictor variables for this study were job dissatisfaction, rotational shift pattern, increased workload, workplace violence, workplace conflicts with colleagues, chronic illness, alcohol uptake, smoking, working hours, and duration of sleep. The selection of predictors in this study is guided by the JD-R Model. These were selected because they align with the job demands and resources framework, where job demands are expected to predict mental health outcomes, and sleep duration serves as a personal resource that may mitigate the effects of high demands. Other psychosocial factors, such as social support and burnout, are acknowledged but not prioritised in this study due to their indirect relationships with the primary job demands in this setting.

### Covariates

Consistent with previous literature [3,21], gender, age, marital status, educational attainment, and occupational role were selected as covariates.

### Data source

Data for the study were obtained using a closed-ended questionnaire prepared and pre-tested (S1 Text). The questionnaire was primarily structured into five sections and administered through face-to-face interviews. The first section (named ‘Section I’) consisted of 8 items and was used to assess the sociodemographic characteristics of the health workers. Likewise, the second section (named ‘Section II’) comprised 10 items and was employed to assess the prevalence of stress. The third section (named ‘Section III’) consisted of 21 items, each describing a specific symptom or attitude related to depression. The fourth section (named ‘Section IV’) also consisted of 21 items and was used to assess the prevalence of anxiety. Furthermore, the fifth section (named ‘Section V’) consisted of 8 items and was used to determine the work-related factors of stress, depression, and anxiety among the health workers.

### Description of the data collection instrument

#### Perceived Stress Scale (PSS-10).

The Perceived Stress Scale (PSS-10) is a psychological instrument for measuring the perception of stress [22]. Developed by Sheldon Cohen and his colleagues in 1983, it is based on the transactional model of stress, which views stress as a product of the interaction between individuals and their environment [23]. This instrument comprises 10 items measured on a 4-point Likert scale, ranging from 1 (Never), 2 (Sometimes), 3 (Often), and 4 (Always) [22,24]. Composite scores were determined to categorise the proportion of health workers with low (≤22), moderate (23 – 35), and high (≥36) stress [3]. Individuals with a moderate to high range (≥23) were considered to have clinically significant stress.

#### Beck’s Depression Inventory (BDI-21).

Beck’s Depression Inventory (BDI-21) is a self-reported measure for assessing the severity of depression [25]. Developed by Aaron T. In 1961, Beck’s instrument consisted of 21 items measured on a 4-point Likert scale, ranging from 1 (Never), 2 (Sometimes), 3 (Often), and 4 (Always) [26]. Composite scores were obtained to categorise the proportion of health workers with minimal (≤21), mild (22 – 27), moderate (28 – 36), and severe (≥37) depression [3]. Health workers with moderate to severe depressive symptoms (≥28) were considered to have clinically significant depression.

#### Beck’s Anxiety Inventory (BAI-21).

Beck’s Anxiety Inventory (BAI-21) is a widely used self-report measure designed to assess the severity of anxiety symptoms in clinical and research settings [27]. Developed by Aaron T. Beck and his colleagues conducted this study in 1988, and it comprised 21 items measured on a 4-point Likert scale, which ranges from 1 (Not at all), 2 (Mildly – but did not bother me much), 3 (Moderately – it was not pleasant at times), and 4 (Severely – it bothered me a lot) [27]. Composite scores were obtained to categorise the proportion of health workers with low (≤21), moderate (22 – 35), and severe (≥36) anxiety [3]. Scores in the moderate to severe range (≥22) indicated clinically meaningful anxiety.

#### Validity and reliability of the data collection instruments.

Although the Perceived Stress Scale (PSS-10), Beck Depression Inventory (BDI-21), and Beck Anxiety Inventory (BAI-21) are well-established tools widely used in Western contexts, it is critical to assess their validity in non-Western populations. This is to ensure their cross-cultural equivalence and construct validity. These instruments have been previously used in Ghana by Opoku Agyemang et al. (2022) and have supported their continued use in Ghanaian populations [3]. For this study, no major modifications were made to the original items. Internal consistency was assessed using McDonald’s omega [ω], with a value of 0.841, 0.919, and 0.940 for PSS-10, BDI-21, and BAI-21, respectively. These also confirmed good reliability for all variables. Also, scores ≥23 on the PSS-10, ≥ 28 on the BDI-21, and ≥22 on the BAI-21 are regarded as clinically significant. This is supported by existing literature, which suggests that individuals with moderate to high levels of stress, depression, and anxiety are at increased risk for impaired functioning and could benefit from clinical interventions [28–30].

### Data management and statistical analysis

Data (S1 Data) from the completed questionnaires were first coded, cleaned, and entered into Microsoft Excel 16 (Microsoft, USA). Subsequently, categorical variables were transformed into frequencies and percentages, whereas continuous variables were summarised as means with their corresponding standard deviations. Following data preparation, the cleaned dataset was exported to STATA version 17 (StataCorp, College Station, TX, USA) for statistical analysis. To begin with, descriptive statistics were used to summarise the sociodemographic and occupational characteristics of the study participants. Next, Pearson’s chi-square tests were employed to assess differences in proportions across categorical variables at a 5% significance level. Thereafter, logistic regression analyses were conducted to examine the association between outcome and predictor variables. In building the regression models, all variables with a p-value ≤ 0.25 in the bivariate logistic regression (Model I) were included in the multivariate logistic regression (Model II). This was necessary to minimise premature exclusion of potential confounders and effect modifiers. Finally, the results of the multivariate model were expressed as adjusted odds ratios (AORs) with corresponding 95% confidence intervals (CIs) and p-values. These estimates were used to identify the independent factors associated with stress, depression, and anxiety among the studied healthcare workers. Statistical significance was maintained at p < 0.05 for all analyses.

### Logistic regression model

In this study, no missing values were recorded; therefore, the logistic regression equation was expressed as a general model.


$$\text{log}\;(p)=\;\text{ln}\left(\frac{p}{\text{1}-p}\right)=\beta_{\text{0}}+\beta_{\text{1}}X_{\text{1}}+\beta_{\text{2}}X_{\text{2}}+\ldots +\beta_{k}X_{k}$$

Where p is the probability of the dependent events occurring, $\beta_{\text{0}}$ is the intercept, and $\beta_{\text{1}},\beta_{\text{2}},\ldots \beta_{k}$ are the coefficients of the independent variables $X_{\text{1}},\;\;X_{\text{2}},\ldots X_{k}$. Model assumptions for the logistic regression were assessed by checking for multicollinearity using variance inflation factors (VIF) and autocorrelation. Multicollinearity was deemed acceptable for all variables, as VIF values were below the threshold. The Durbin-Watson statistic was applied to assess the independence of residuals. The analysis produced a value of 1.946. This result indicates no evidence of autocorrelation, as it falls within the acceptable range of 1.5 to 2.5.

### Assessment of model fitness

To assess the fitness of the logistic regression models, several diagnostic indices were considered. Initially, the likelihood ratio chi-square (LR χ^2^) test evaluated the overall significance of the models. Subsequently, pseudo-R² values were examined to determine the proportion of variance explained. To further assess the fitness of the model, the Hosmer-Lemeshow test was conducted, with non-significant results indicating adequate model performance. In addition, Receiver Operating Characteristic (ROC) curve analysis evaluated the models’ discriminative ability. Finally, the precision of coefficient estimates was examined through their standard errors.

### Baises and mitigation strategies

Sampling bias may arise from differences in the populations of the selected units within the hospital. To address this, a quota proportional to the sample size was allotted to each unit, ensuring representativeness. Meanwhile, selection bias was minimised through randomisation, giving every health worker in the respective units an equal chance of being included in the sample.

## Results

### Sociodemographic characteristics of health workers

The estimated sample size for the study was 324 health workers sampled from 6 units within the health facility. Of these, 316 consented to participate, indicating a response rate of 97.53%. Hence, the analyses were based on data from 316 health workers. **Table 2** presents the sociodemographic characteristics of the health workers. The majority (57.9%) of the participants were females, 41.5% were between 18 and 25 years old, and 9.6% were above 41 years old. In addition, 60.4% of the participants were single, whereas 2.2% were cohabitating. The educational status ranges from Senior High School (5.7%) to Bachelor’s Degree (29.4%). The majority of the health workers (50.6%) were Christians, 76.6% were nurses, 11.0% were Allied Health Personnel, and 1.9% were Dispensary Technicians. The majority of the participants (65.8%) had worked for between 1 and 7 years; 30.1% earned Ghȼ3000 and above, 28.2% earned less than Ghȼ1500, and 25% earned between Ghȼ1500 and Ghȼ2000 monthly.

**Table 2:** Socio-demographic characteristics of health workers.

Variable	Frequency (N)	Percentage (%)
Gender		
Male	133	42.1
Female	183	57.9
Age		
18-25	131	41.5
26-30	91	28.8
31-40	63	19.9
41+	31	9.6
Marital status		
Single	191	60.4
Married	118	37.3
Cohabitation	7	2.2
Education		
SHS	18	5.7
Certificate	102	32.3
Diploma	103	32.6
Bachelor’s degree	93	29.4
Religious affiliation		
Islam	144	45.6
Christianity	160	50.6
Traditional religion	12	3.8
Occupational category		
Nurses	242	76.6
Allied Health Personnel	35	11
Physicians	17	5.4
Ambulance Personnel	10	3.2
Dispensary Technicians	6	1.9
Orderlies	6	1.9
Number of years in the health profession		
1-7 years	208	65.8
8-12 years	59	18.7
13-17 years	35	11.1
18-23 years	14	4.4
Monthly income		
Less than Ghȼ 1500	89	28.2
Ghȼ 1500–2000	79	25
Ghȼ 2100–2900	53	16.8
Ghȼ 3000 and above	95	30.1

### Prevalence of stress, depression, and anxiety among healthcare workers

**Table 3** presents the frequency distribution of participants’ responses to items on the Perceived Stress Scale (PSS-10), Beck Depression Inventory (BDI-21), and Beck Anxiety Inventory (BAI-21). The majority of the health workers emphasised “Always” and “Often” to the various items (**Table 3**). Based on the composite scores from the PSS-10, a combined 66.5% (95% CI: 61.20 – 71.70) of individuals were found to have clinically significant stress, with 36.1% reporting high stress and 30.4% moderate stress levels (**Table 4**). Similarly, from the composite scores from the Beck Depression Inventory (BDI-21), the prevalence of clinically significant depression was 63.6% (95% CI: 58.30 – 68.90). This category includes 32.3% of patients experiencing severe depression and 31.3% moderate (**Table 4**). Finally, assessment using the composite scores from the Beck Anxiety Inventory (BAI-21) indicated that clinically significant anxiety affected 87.9% (95% CI: 84.40 – 91.60) of health workers, with 35.4% experiencing severe anxiety and 52.5% moderate levels (**Table 4**).

**Table 3:** Frequency distribution of participant responses to Perceived Stress Scale, Beck’s Depression Inventory, and Beck’s Anxiety Inventory.

Perceived Stress Scale (PSS-10)	Scale							
	Always		Often		Sometimes		Never	
Items	N	%	N	%	N	%	N	%
Upset because of something that happened unexpectedly?	4	1.3	17	5.4	208	65.8	87	27.5
You were unable to control the important things in your life?	8	2.5	12	3.8	205	64.9	91	28.8
Nervous and stressed?	10	3.2	31	9.8	224	70.9	51	16.1
You could not cope with all the things that you had to do?	3	0.9	14	4.4	225	71.2	74	23.4
Angry because of things outside of your control?	14	4.4	20	6.3	196	62	86	27.2
Difficulties were piling up so high that you could not overcome them.	14	4.4	18	5.7	180	57	104	32.9
Confident about your ability to handle your problems?	91	28.8	47	14.9	133	42.1	45	14.2
Things were going your way?	40	12.7	48	15.2	186	58.9	42	13.3
Able to control irritations in your life?	54	17.1	45	14.2	165	52.2	52	16.5
You were on top of things?	55	17.4	35	11.1	165	52.2	61	19.3
Beck’s Depression Inventory	**Scale**							
	**Always**		**Often**		**Sometimes**		**Never**	
Items	**N**	**%**	**N**	**%**	**N**	**%**	**N**	**%**
You are so unhappy that you can’t stand it	5	1.6	18	5.7	176	55.7	117	37
You feel the future is hopeless and that things cannot improve	6	1.9	20	6.3	116	36.7	174	55.1
You feel you are a complete failure as a person	8	2.5	12	3.8	97	30.7	199	63
You are dissatisfied or bored with everything	8	2.5	7	2.2	155	49.1	146	46.2
You feel guilty all of the time	6	1.9	17	5.4	127	40.2	166	52.5
You feel you are being punished	7	2.2	13	4.1	128	40.5	168	53.2
You hate yourself	4	1.3	8	2.5	94	29.7	210	66.5
You blame yourself for everything bad that happens	7	2.2	18	5.7	150	47.5	141	44.6
You would kill yourself if you had the chance	3	0.9	7	2.2	71	22.5	235	74.4
You used to cry, but now you can’t cry, even though you want to	12	3.8	17	5.4	153	48.4	134	42.4
You feel irritated all the time	6	1.9	18	5.7	143	45.3	149	47.2
You have lost all interest in other people	14	4.4	21	6.6	131	41.5	150	47.5
You can’t make decisions at all anymore	5	1.6	18	5.7	121	38.3	172	54.4
You believe that you look ugly	3	0.9	11	3.5	79	25	223	70.6
You can’t do any work at all	6	1.9	10	3.2	93	29.4	207	65.5
You wake up several hours earlier and cannot get back to sleep	13	4.1	14	4.4	197	62.3	92	29.1
You are too tired to do anything	6	1.9	16	5.1	189	59.8	105	33.2
You have no appetite at all anymore	9	2.8	16	5.1	165	52.2	126	39.9
You have lost significant weight	6	1.9	21	6.6	158	50	131	41.5
You have lost interest in sex completely	11	3.5	13	4.1	149	47.2	143	45.3
Beck’s Anxiety Inventory	**Scale**							
	**Severely**		**Moderately**		**Mildly**		**Never**	
Items	**N**	**%**	**N**	**%**	**N**	**%**	**N**	**%**
Numbness or tingling	15	4.7	42	13.3	121	38.3	138	43.7
Feeling hot	12	3.8	59	18.7	124	39.2	121	38.3
Wobbliness in legs	10	3.2	40	12.7	96	30.4	170	53.8
Unable to relax	12	3.8	46	14.6	114	36.1	144	45.6
Fear of the worst happening	28	8.9	42	13.3	119	37.7	127	40.2
Dizzy or lightheaded	20	6.3	33	10.4	112	35.4	151	47.8
Heat pounding/racing	10	3.2	47	14.9	102	32.3	157	49.7
Shaky/unsteady	6	1.9	40	12.7	107	33.9	163	51.6
Terrified or afraid	8	2.5	38	12	117	37	153	48.4
Nervous	14	4.4	42	13.3	130	41.1	130	41.1
Feeling of choking	7	2.2	33	10.4	85	26.9	191	60.4
Hands trembling	6	1.9	34	10.8	99	31.3	177	56
Fear of losing control	15	4.7	39	12.3	97	30.7	165	52.2
Difficulty in breathing	4	1.3	27	8.5	75	23.7	210	66.5
Fear of dying	33	10.4	36	11.4	81	25.6	166	52.5
Scared	10	3.2	42	13.3	109	34.5	155	49.1
Indigestion	10	3.2	33	10.4	105	33.2	168	53.2
Faint/lightheaded	7	2.2	23	7.3	85	26.9	201	63.6
Face flushed	8	2.5	27	8.5	101	32	180	57
Hot/cold sweats	15	4.7	48	15.2	119	37.7	134	42.4

N, Frequency; %, Percentage.

**Table 4:** Mental health status according to categories and thresholds.

Mental Health Issue	Category	Score Range (Threshold)	Frequency (N)	Prevalence (%)
Stress	High	≥36	114	36.1
	Moderate	23–35	96	30.4
	Low	≤22	106	33.5
Mean ± SD	2.03 ± 0.83			
Stress based on threshold	**Total number of healthcare workers with clinically significant stress**	**≥23 (Moderate + High)**	**210**	**66.5**
Depression	Severe	≥37	102	32.3
	Moderate	28–36	99	31.3
	Mild	22–27	77	24.4
	Minimal	≤21	38	12.0
Mean ± SD	2.84 ± 1.01			
Depression based on threshold	**Total number of healthcare workers with clinically significant depression**	**≥28 (Moderate + Severe)**	**201**	**63.6**
Anxiety	Severe	≥36	112	35.4
	Moderate	22–35	166	52.5
	Low	≤21	38	12.0
Mean ± SD	2.23 ± 0.64			
Anxiety based on threshold	**Total number of healthcare workers with clinically significant anxiety**	**≥22 (Moderate + Severe)**	**278**	**87.9**

N, Frequency; %, Percentage; ≥ , Greater than or equal to; ≤ , Less than or equal to.

### Factors associated with stress, depression, and anxiety among health workers

Tables 5–7 present the bivariate and multivariate analyses of correlates of stress, depression, and anxiety among health workers at Kintampo North Municipal Hospital

**Table 5:** Factors associated with stress among health workers.

		Stress based on threshold (≥23)	Model I			Model II		
Items	N [%]	[n] [% (95%CI)]	COR	[95%CI]	p-value	AOR	[95%CI]	p-value
Job dissatisfaction								
Yes	270 [85.4]	177 [65.6% (59.9 – 71.2)]	3.33	[1.12 - 5.91]	0.033*	5.52	[2.13 - 7.96]	0.037*
No	46 [14.6]	33 [71.7% (58.7 – 84.8)]	1			1		
Rotational shift pattern								
Yes	233 [73.7]	164 [70.4% (64.6 – 76.3)]	2.29	[1.13 - 4.66]	0.003*	4.55	[2.17 - 6.81]	0.034*
No	83 [26.3]	46 [55.4% (44.7 – 66.1)]	1			1		
Increase in workload								
Yes	160 [50.6]	99 [61.9% (54.3 – 69.4)]	5.37	[2.45 - 7.80]	< 0.001*	3.18	[1.04 - 5.03]	0.042*
No	156 [49.4]	111 [71.2% (64.0 – 78.3)]	1			1		
Workplace violence in the past 1 month								
Yes	108 [34.2]	71 [65.7% (56.8 – 74.7)]	2.84	(0.31 - 5.24)	0.729	2.91	[0.48 - 3.73]	0.793
No	208 [65.8]	139 [66.8% (60.4 – 73.2)]	1			1		
Conflicts with colleagues in the past 1 month								
Yes	79 [25]	51 [64.6% (54.0 – 75.1)]	4.92	[0.53 - 7.60]	0.778	3.75	[0.37 - 5.50]	0.427
No	237 [75]	159 [67.1% (61.1 – 73.1)]	1			1		
Chronic illness								
Yes	63 [19.9]	35 [55.6% (43.3 – 67.8)]	1.35	[1.12 - 3.23]	0.024*	1.52	[1.31 - 3.14]	0.028*
No	253 [80.1]	175 [69.2% (63.5 – 74.9)]	1			1		
Alcohol uptake								
Yes	38 [12]	25 [65.8% (50.7 – 80.9)]	1.46	[1.06 - 3.23]	0.033*	1.41	[1.16 - 3.55]	0.043*
No	278 [88]	185 [66.6% (61.0 – 72.1)]	1			1		
Smoking								
Yes	22 [7]	16 [72.7% (54.1 – 91.3)]	1.17	[0.44 - 3.10]	0.743	1.1	[0.35 - 3.45]	0.867
No	294 [93]	194 [66.0 (60.6 – 71.4)]	1			1		
Working hours per day								
Above 8 hours	222 [70.3]	147 [66.2% (60.0 – 72.4)]	1.47	[1.28 - 3.17]	0.032*	2.34	[1.13 - 3.05]	0.046*
Between 6–7 hours	61 [19.3]	41 [67.2% (55.5 – 79.0)]	1.01	[1.00 - 2.44]	0.009*	2.86	[1.33 - 4.17]	0.048*
Less than 6 hours	33 [10.4]	22 [66.7% (50.6 – 82.8)]	1			1		
Sleep hours per day								
5 hours	89 [28.2]	49 [55.1% (44.7 – 65.4)]	1.24	[1.12 - 2.53]	0.038*	2.97	[1.51 - 4.85]	0.014*
6 - 7 hours	166 [52.5]	120 [72.3% (65.5 – 79.1)]	1.09	[1.01 - 2.06]	0.016*	1.91	[1.46 - 2.23]	0.017*
8 hours	61 [19.3]	41 [67.2% (55.4 – 79.0)]	1			1		
Gender								
Male	133 [42.1]	89 [66.9% (58.9 – 74.9)]	1.68	1.14 - 2.73	0.034*	3.89	1.92 - 5.10	0.028*
Female	183 [57.9]	121 [66.1% (59.3 – 73.0)]	1			1		
Occupational category								
Nurses	242 [76.6]	176 [72.7% (67.1 – 78.3)]	4.99	1.24 - 6.14	0.029*	5.77	1.56 - 7.73	0.026*
Allied Health Personnel	35 [11]	15 [42.9% (26.5 – 59.2)]	2.15	1.21 - 3.41	0.034*	2.12	1.21 - 3.86	0.041*
Physicians	17 [5.4]	8 [47.1% (23.3 – 70.8)]	4.17	2.13 - 6.19	0.027*	3.14	1.31 - 5.96	0.048*
Emergency Medical Technicians	10 [3.2]	6 [60.0% (29.6 – 90.4)]	3.11	1.44 - 5.39	0.039*	3.71	1.37 - 5.41	0.013*
Dispensary technicians	6 [1.9]	3 [50.0% (10.0 – 90.0)]	5.16	2.71 - 7.51	0.028*	2.31	1.14 - 3.60	0.028*
Orderlies	6 [1.9]	2 [33.3% (27.0 – 71.1)]	1			1		

[LR χ2 (10) = 226.8, Prob > χ2 = 0.001, Pseudo R^2^ = 0.7729, Hosmer–Lemeshow χ2 (8) = 7.87, Prob> χ2 = 0.4467, ROC = 0.91 [0.87 - 0.95], SE = 0.018

N, Frequency; %, Percentage; CI, Confidence interval; COR, Crude odds ratio; AOR, Adjusted odds ratio; *, p < 0.05, ROC: Receiver operating characteristic.

**Table 6:** Factors associated with depression among health workers.

		Depression based on threshold (≥23)	Model I			Model II		
Items	N [%]	[n] [% (95%CI)]	COR	[95%CI]	p-value	AOR	[95%CI]	p-value
Job dissatisfaction								
Yes	270 [85.4]	165 [61.1% (55.2 – 66.7)]	3.99	[1.50 - 5.96]	0.013*	4.95	[2.45 - 6.01]	0.018*
No	46 [14.6]	36 [78.3% (64.4 – 87.7)]	1			1		
Rotational shift pattern								
Yes	233 [73.7]	142 [60.9% (54.6 – 67.0)]	2.29	[1.73 - 4.25]	0.032*	3.22	[1.70 - 5.38]	0.014*
No	83 [26.3]	59 [71.1% (60.6 – 79.7)]	1			1		
Increase in workload								
Yes	160 [50.6]	115 [71.9% (64.5 – 78.3)]	2.85	[1.52 - 4.88]	0.041*	4.81	[1.49 - 7.45]	0.042*
No	156 [49.4]	86 [55.1% (47.3 – 62.7)]	1			1		
Workplace violence in the past 1 month								
Yes	108 [34.2]	81 [75.0% (66.1 – 82.2)]	1.01	[0.66 - 1.67]	0.961	1.08	[0.57 - 2.05]	0.793
No	208 [65.8]	120 [57.7% (50.9 – 64.2)]	1			1		
Conflicts with colleagues in the past 1 month								
Yes	79 [25]	64 [81.0% (71.0 – 88.1)]	1.08	[0.62 - 1.87]	0.778	1.32	[0.66 - 2.63]	0.427
No	237 [75]	137 [57.8% (51.4 – 63.9)]	1			1		
Chronic illness								
Yes	63 [19.9]	47 [74.6% (62.7 – 83.7)]	1.73	[1.39 - 3.37]	0.024*	2.65	[1.31 - 3.36]	0.028*
No	253 [80.1]	154 [60.9% (54.7 – 66.7)]	1			1		
Alcohol uptake								
Yes	38 [12]	26 [68.4% (52.5 – 80.9)]	1.68	[1.30 - 3.53]	0.034*	3.72	[1.28 - 5.76]	0.043*
No	278 [88]	175 [62.9% (57.1 – 68.4)]	1			1		
Smoking								
Yes	22 [7]	21 [95.5% (78.2 – 99.2)]	0.85	[0.32 - 2.24]	0.743	0.9	[0.28 - 2.84]	0.867
No	294 [93]	180 [61.2% (55.5 – 66.6)]	1			1		
Working hours per day								
Above 8 hours	222 [70.3]	140 [63.1% (56.5 – 69.1)]	1.67	[1.31 - 3.46]	0.032*	2.74	[1.32 - 3.68]	0.046*
Between 6–7 hours	61 [19.3]	39 [63.9% (51.4 – 74.8)]	1.98	[1.40 - 2.38]	0.009*	2.16	[1.45 - 3.95]	0.048*
Less than 6 hours	33 [10.4]	22 [66.7% (49.6 – 80.2)]	1			1		
Sleep hours per day								
5 hours	89 [28.2]	64 [71.9% (61.8 – 80.2)]	1.8	[1.39 - 2.62]	0.038*	5.02	[1.53 - 7.93]	0.039*
6 - 7 hours	166 [52.5]	104 [62.7% (55.1 – 69.6)]	1.9	[1.48 - 1.70]	0.016*	3.98	[1.42 - 5.16]	0.037*
8 hours	61 [19.3]	33 [54.1% (41.7 – 66.0)]	1			1		
Gender								
Male	133 [42.1]	77 [57.9% (49.4 – 65.9)]	1.43	1.30 - 2.70	0.048*	1.55	1.15 - 3.04	0.033*
Female	183 [57.9]	124 [67.8% (60.7 – 74.1)]	1			1		
Occupational category								
Nurses	242 [76.6]	162 [66.9% (60.8 – 72.6)]	1.87	1.28 - 2.66	0.018*	1.82	1.25 - 2.67	0.048*
Allied Health Personnel	35 [11]	17 [48.6% (33.0 – 64.4)]	1.46	1.29 - 3.10	0.015*	1.34	1.29 - 3.70	0.041*
Physicians	17 [5.4]	10 [58.8% (36.0 – 78.4)]	1.61	1.16 - 2.30	0.044*	1.66	1.14 - 2.92	0.046*
Emergency Medical Technicians	10 [3.2]	5 [50.0% (23.7 – 76.3)]	1.16	1.03 - 0.84	0.031*	1.15	1.02 - 2.88	0.036*
Dispensary Technicians	6 [1.9]	3 [50.0% (18.8 – 81.2)]	1.43	1.15 - 2.23	0.019*	1.38	1.12 - 2.34	0.019*
Orderlies	6 [1.9]	4 [66.7% (30.0 – 90.3)]	1			1		

[LR χ2 (10) = 214.8, Prob > χ2 = 0.001, Pseudo R2 = 0.7229, Hosmer–Lemeshow χ2 (8) = 10.99, Prob> χ2 = 0.2023, ROC = 0.8722 [0.81 - 0.93], SE = 0.0285

N, Frequency; %, Percentage; CI, Confidence interval; COR, Crude odds ratio; AOR, Adjusted odds ratio; *, p < 0.05

**Table 7:** Factors associated with anxiety among health workers.

		Anxiety based on threshold (≥23)	Model I			Model II		
Items	N [%]	[n] [% (95%CI)]	COR	[95%CI]	p-value	AOR	[95%CI]	p-value
Job dissatisfaction								
Yes	270 [85.4]	233 [86.3% (81.7 – 89.9)]	2.54	[1.31 - 4.91]	0.023*	2.51	[1.28 - 3.95]	0.034*
No	46 [14.6]	45 [97.8% (88.7 – 99.6)]	1			1	1	
Rotational shift pattern								
Yes	233 [73.7]	204 [87.6% (82.7 – 91.2)]	3.11	[1.35 - 7.17]	0.007*	3.24	[1.31 - 4.01]	0.011*
No	83 [26.3]	74 [89.2% (80.7 – 94.2)]	1			1	1	
Increase in workload								
Yes	160 [50.6]	152 [95.0% (90.4 – 97.4)]	2.99	[1.57 - 4.17]	0.019*	2.32	[1.69 - 4.53]	0.037*
No	156 [49.4]	126 [80.8% (73.9 – 86.2)]	1			1	1	
Workplace violence in the past 1 month								
Yes	108 [34.2]	101 [93.5% (87.2 – 96.8)]	0.49	[0.20 - 1.20]	0.122	0.66	[0.23 - 1.93]	0.458
No	208 [65.8]	177 [85.1% (79.6 – 89.3)]	1			1	1	
Conflicts with colleagues in the past 1 month								
Yes	79 [25]	75 [94.9% (87.7 – 98.0)]	0.62	[0.31 - 1.23]	0.173	0.66	[0.29 - 1.48]	0.317
No	237 [75]	203 [85.7% (80.6 – 89.5)]	1			1	1	
Chronic illness								
Yes	63 [19.9]	61 [96.8% (89.1 – 99.1)]	2.31	[1.12 - 3.83]	0.019*	2.44	[1.14 - 4.30]	0.014*
No	253 [80.1]	217 [85.8% (80.9 – 89.5)]	1			1	1	
Alcohol uptake								
Yes	38 [12]	38 [100.0% (90.8 – 100.0)]	1.42	[1.21 - 2.84]	0.015*	1.55	[1.22 - 2.33]	0.019*
No	278 [88]	240 [86.3% (81.8 – 89.9)]	1			1	1	
Smoking								
Yes	22 [7]	22 [100.0% (85.1 – 100.0)]	1.32	[0.56 - 3.31]	0.517	0.95	[0.36 - 2.53]	0.929
No	294 [93]	256 [87.1% (82.8 – 90.4)]	1			1	1	
Working hours per day								
Above 8 hours	222 [70.3]	192 [86.5% (81.4 – 90.4)]	2.77	[1.32 - 4.82]	0.047*	2.56	[1.21 - 4.53]	0.026*
Between 6–7 hours	61 [19.3]	55 [90.2% (80.2 – 95.4)]	2.42	[1.13 - 4.24]	0.014*	2.37	[1.11 - 4.30]	0.012*
Less than 6 hours	33 [10.4]	31 [93.9% (80.4 – 98.3)]	1			1	1	
Sleep hours per day								
5 hours	89 [28.2]	83 [93.3% (86.1 – 96.9)]	2.26	[1.05 - 4.90]	0.037*	2.17	[1.95 - 4.94]	0.043*
6 - 7 hours	166 [52.5]	145 [87.3% (81.4 – 91.6)]	3.08	[1.26 - 5.48]	0.013*	3.23	[1.26 - 5.24]	0.014*
8 hours	61 [19.3]	50 [82.0% (70.5 – 89.6)]	1			1	1	
Gender								
Male	133 [42.1]	113 [85.0% (77.9 – 90.0)]	1.22	1.01 - 2.33	0.034*	3.22	1.22 - 5.23	0.041*
Female	183 [57.9]	165 [90.2% (85.0 – 93.7)]	1			1		
Occupational category								
Nurses	242 [76.6]	225 [93.0% (89.0 – 95.6)]	8.26	1.70 - 10.32	0.033*	9.51	1.62 - 14.97	0.015*
Allied Health Personnel	35 [11]	28 [80.0% (64.1 – 90.0)]	6.26	1.35 - 8.30	0.021*	7.84	1.36 - 10.89	0.019*
Physicians	17 [5.4]	14 [82.4% (59.0 – 93.8)]	3.03	1.34 - 5.62	0.035*	1.72	1.14 - 2.58	0.026*
Emergency Medical Technicians	10 [3.2]	6 [60.0% (31.3 – 83.2)]	1.73	1.17 - 3.07	0.027*	1.43	1.23 - 2.41	0.031*
Dispensary technicians	6 [1.9]	2 [33.3% (9.7 – 70.0)]	2.24	1.26 - 4.99	0.041*	2.48	1.25 - 4.13	0.044*
Orderlies	6 [1.9]	3 [50.0% (18.8 – 81.2)]	1			1		

[LR χ2 (10) = 262.8, Prob > χ2 = 0.001, Pseudo R2 = 0.8335, Hosmer–Lemeshow χ2 (8) = 4.53, Prob > χ2 = 0.8067, ROC = 0.9067 [0.87 - 0.94], SE = 0.0817N, Frequency; %, Percentage; CI, Confidence interval; COR, Crude odds ratio; AOR, Adjusted odds ratio; *, p < 0.05

#### Stress (Based on the threshold).

**Fig 3:**
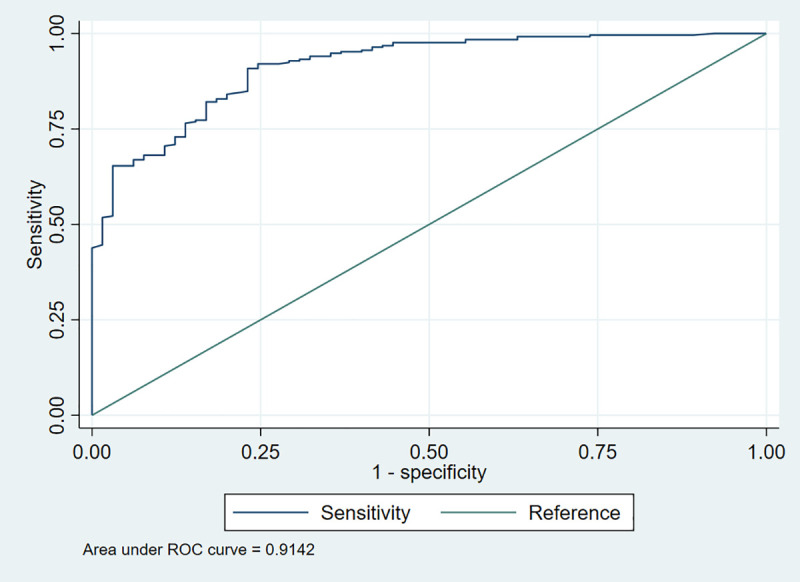
Receiver operating characteristic (ROC) curve for stress.

For stress, the adjusted model (**Model II**) was statistically significant [LR χ^2^ (10) = 226.8, *p* < 0.001] and accounted for approximately 77.3% of the variation in the outcome [pseudo-R^2^ = 0.773]. In addition, the model demonstrated good fit and predictive performance, with a high ROC value of 0.9142 (**Fig 3**), and no evidence of poor fit based on the Hosmer-Lemeshow test [χ^2^(8) = 7.87, p = 0.4467]. The standard error of the model estimates was low (0.0182), further supporting the reliability of the findings. After adjusting [**Model II**], several predictors remained meaningfully associated with higher odds of stress (**Table 5**). Health workers reporting job dissatisfaction had about 4.6 times higher odds of experiencing stress compared with those satisfied with their job (AOR = 4.55, 95% CI: 2.17 – 6.81, p = 0.034). Likewise, those working under a rotational shift pattern had 4.55 times the odds of being stressed compared with those on fixed shifts (AOR = 4.55; 95% CI: 2.17 – 6.81, p = 0.034). Similarly, health workers reporting increased workload had 3.18 times the odds of experiencing stress compared to those with no reported increase in workload (AOR = 3.18, 95% CI: 1.04 –5.03, p = 0.042). Also, health workers with chronic illnesses had 1.52 times the odds of being stressed compared to those without chronic illness (AOR = 1.52, 95% CI: 1.31 – 3.14, p = 0.028). Furthermore, alcohol uptake was also significantly associated with stress, such that health workers who consumed alcohol had 1.41 times the odds of stress compared to non-drinkers (AOR = 1.41, 95% CI: 1.16 – 3.55, p = 0.043).

Moreover, health workers who worked more than 8 hours per day had 2.34 times the odds of being stressed compared with those who worked less than 6 hours (AOR = 2.34, 95% CI: 1.13 – 3.05, p = 0.046). Similarly, those working between 6 and 7 hours had 2.86 times the odds of stress relative to those working for less than 6 hours (AOR = 2.86, 95% CI: 1.33 – 4.17, p = 0.048). In addition, sleep duration also showed significance. Workers sleeping only 5 hours per day had 2.97 times the odds of experiencing stress compared to those sleeping 8 hours (AOR = 2.97, 95% CI: 1.51 – 4.85, p = 0.014). Those sleeping between 6 and 7 hours also had increased odds of stress (AOR = 1.91, 95% CI: 1.46 – 2.23, p = 0.017). Gender was a significant determinant, with male health workers having 3.89 times the odds of stress compared to female counterparts (AOR = 3.89, 95% CI: 1.92 – 5.10, p = 0.028). Likewise, occupational roles significantly influenced the odds of experiencing stress. Compared with orderlies, nurses had 5.77 times the odds of experiencing stress (AOR = 5.77, 95% CI: 1.56–7.73, p = 0.026), whereas emergency medical technicians had 3.71 times the odds (AOR = 3.71, 95% CI: 1.37–5.41, p = 0.013). Allied health personnel (AOR = 2.12, 95% CI: 1.21–3.86, p = 0.041) and physicians (AOR = 3.14, 95% CI: 1.31–5.96, p = 0.048) also showed significantly higher odds of experiencing stress compared to orderlies.

#### Depression (Based on the threshold).

**Fig 4:**
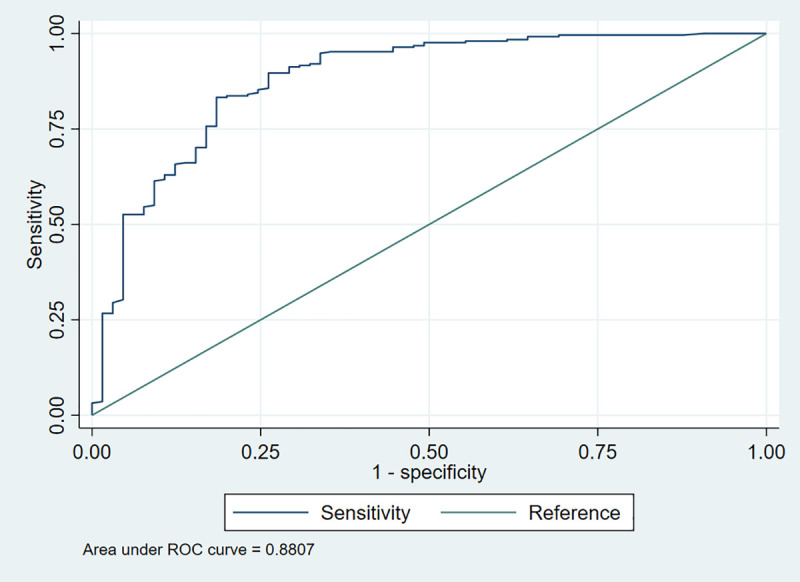
Receiver operating characteristic (ROC) curve for depression.

For depression, the adjusted model (**Model II**) was statistically significant [LR χ^2^ (10) = 214.8, *p* < 0.001] and accounted for approximately 72.3% of the variation in the outcome [pseudo-R^2^ = 0.723]. Likewise, the model demonstrated good fit, with a ROC value of 0.8722 (**Fig 4**), suggesting strong predictive performance. The Hosmer-Lemeshow test [χ^2^(8) = 10.99, p = 0.2023] did not indicate any significant departure from a good fit. The standard error of the model estimates was relatively low (0.0285), further supporting the robustness of the findings. From **Table 6**, health workers who reported job dissatisfaction had more than 4.95 times the odds of experiencing depression compared to those satisfied with their job (AOR = 4.95, 95% CI: 2.45 – 6.01, p = 0.018). Similarly, working under a rotational shift pattern was associated with increased odds of depression, with affected workers having 3.22 times the odds compared to those on fixed shifts (AOR = 3.22, 95% CI: 1.70 – 5.38, p = 0.014). In addition, reporting an increase in workload was significantly linked to higher odds of depression. Health workers who experienced increased workload had 4.81 times the odds of being depressed compared with those who did not (AOR = 4.81, 95% CI: 1.49 – 7.45, p = 0.042). Likewise, the presence of chronic illness was a notable factor; those with chronic conditions had 2.65 times greater odds of experiencing depression than their healthier counterparts (AOR = 2.65, 95% CI: 1.31 – 3.36, p = 0.028). Moreover, alcohol use was significantly associated with depression. Health workers who consumed alcohol had 3.72 times the odds of being depressed compared with non-drinkers (AOR = 3.72, 95% CI: 1.28 – 5.76, p = 0.043). Conversely, smoking was not found to be a significant predictor of depression (AOR = 0.90, 95% CI: 0.28 – 2.84, p = 0.867).

Working hours also played a crucial role. Those working more than 8 hours per day had 2.74 times the odds of experiencing depression relative to those working less than 6 hours (AOR = 2.74, 95% CI: 1.32 – 3.68, p = 0.046). Additionally, workers with 6–7-hour shifts also showed increased odds (AOR = 2.16, 95% CI: 1.45 – 3.95, p = 0.048). Furthermore, shorter sleep duration was strongly associated with depression. Health workers who slept for only 5 hours per day had 5.02 times the odds of being depressed compared with those who slept for 8 hours (AOR = 5.02, 95% CI: 1.53 – 7.93, p = 0.039). Those who slept for 6–7 hours similarly had elevated odds (AOR = 3.98, 95% CI: 1.42 – 5.16, p = 0.037). Gender also emerged as a significant determinant. Male healthcare workers had 1.55 times higher odds of depression than female healthcare workers (AOR = 1.55, 95% CI: 1.15 – 3.04, p = 0.033). Finally, the odds of depression varied notably across roles. Compared with orderlies, nurses exhibited 1.82 times the odds of experiencing depression (AOR = 1.82, 95% CI: 1.25 – 2.67, p = 0.048). In the same vein, emergency medical technicians had elevated odds (AOR = 1.15, 95% CI: 1.02 – 2.88, p = 0.036), as did allied health personnel (AOR = 1.34, 95% CI: 1.29 – 3.70, p = 0.041), physicians (AOR = 1.66, 95% CI: 1.14 – 2.92, p = 0.046), and dispensary technicians (AOR = 1.38, 95% CI: 1.12 – 2.34, p = 0.019).

#### Anxiety (Based on the threshold).

**Fig 5:**
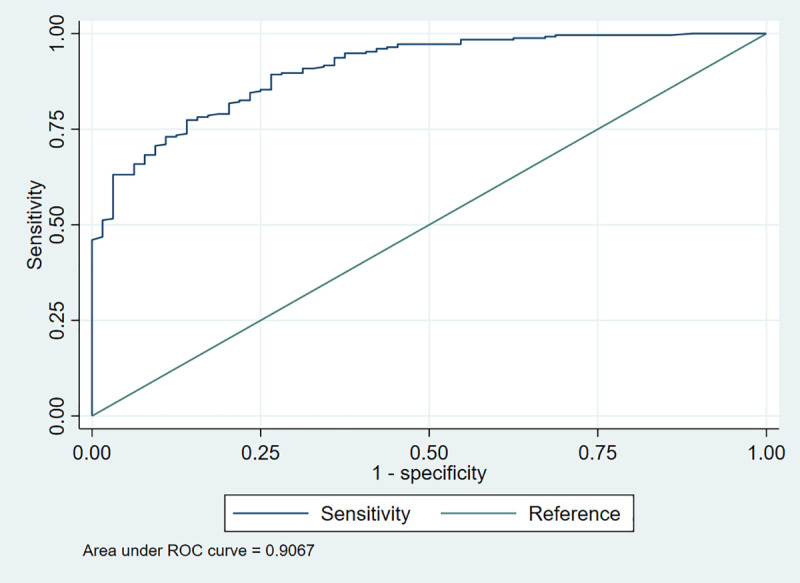
Receiver operating characteristic (ROC) curve for anxiety.

For anxiety, the adjusted model (**Model II**) was statistically significant [LR χ^2^ (10) = 262.8, *p* < 0.001] and accounted for approximately 83.4% of the variation in the outcome [pseudo-R^2^ = 0.83.4]. Similarly, the model also demonstrated strong predictive performance with a high ROC value of 0.9067 (**Fig 5**). The Hosmer-Lemeshow test [χ^2^(8) = 4.53, p = 0.8067] suggested a good fit, as there was no significant evidence of poor model fit. The standard error of the model estimates was relatively low (0.0817), further confirming the reliability of the findings. As presented in **Table 7**, dissatisfied workers had 2.51 times the odds of experiencing anxiety (AOR = 2.51, 95% CI: 1.28 – 3.95, p = 0.034). Likewise, working under a rotational shift pattern was significantly associated with increased anxiety. Those working rotational shifts had 3.24 times the odds of experiencing anxiety compared with those with fixed shifts (AOR = 3.24, 95% CI: 1.31 – 4.01, p = 0.011). In addition, an increase in workload significantly predicted anxiety. Health workers who reported increased workload had 2.32 times the odds of anxiety relative to those who did not report an increase (AOR = 2.32, 95% CI: 1.69–4.53, p = 0.037). Moreover, the presence of a chronic illness emerged as a significant factor. Those with chronic health conditions had 2.44 times the odds of anxiety relative to their healthier counterparts (AOR = 2.44, 95% CI: 1.14 – 4.30, p = 0.014). Furthermore, alcohol use was significantly associated with anxiety, such that health workers who consumed alcohol had 1.55 times the odds of experiencing anxiety compared with non-drinkers (AOR = 1.55, 95% CI: 1.22 – 2.33, p = 0.019).

In addition, working hours per day significantly influenced anxiety levels. Those working more than 8 hours per day had 2.56 times the odds of anxiety compared with those working less than 6 hours (AOR = 2.56, 95% CI: 1.21 – 4.53, p = 0.026). Similarly, those who worked between 6 and 7 hours had 2.37 times the odds of experiencing anxiety (AOR = 2.37, 95% CI: 1.11 – 4.30, p = 0.012). Sleep duration was another important determinant. Health workers who reported sleeping for only 5 hours per day had 2.17 times the odds of anxiety compared with those who slept for 8 hours (AOR = 2.17, 95% CI: 1.95 – 4.94, p = 0.043). Likewise, those sleeping between 6 and 7 hours had 3.23 times the odds (AOR = 3.23, 95% CI: 1.26 – 5.24, p = 0.014). Gender was also significantly associated with anxiety. Male healthcare workers had 3.22 times the odds of experiencing anxiety compared to their female counterparts (AOR = 3.22, 95% CI: 1.22 – 5.23, p = 0.041). Finally, occupational roles played a notable role in predicting anxiety. Compared with orderlies, nurses had 9.51 times the odds of anxiety (AOR = 9.51, 95% CI: 1.62 – 14.97, p = 0.015), whereas allied health personnel had 7.84 times the odds (AOR = 7.84, 95% CI: 1.36 – 10.89, p = 0.019). Physicians also showed increased odds (AOR = 1.72, 95% CI: 1.14 – 2.58, p = 0.026), as did emergency medical technicians (AOR = 1.43, 95% CI: 1.23 – 2.41, p = 0.031) and dispensary technicians (AOR = 2.48, 95% CI: 1.25 – 4.13, p = 0.044).

## Discussion

### Prevalence of stress, depression, and anxiety among healthcare workers

This study assessed the prevalence of stress, depression, and anxiety among health workers in the Kintampo North Municipal Hospital. It revealed that 66.5% (95% CI: 61.20 – 71.70), 63.6% (95% CI: 58.30 – 68.90), and 87.9% (95% CI: 84.40 – 91.60) of the health workers experienced stress, depression, and anxiety, respectively. These figures align with previous reports, which have similarly documented high levels of psychological distress among healthcare professionals in comparable low- and middle-income contexts [3,21,31–34]. The convergence of these findings underlines the persistent vulnerability of healthcare workers to mental health challenges, despite global recognition of the critical role they play in health systems.

In high-income countries, increasing attention has been devoted to workplace wellness initiatives, structured mental health support services, and policies aimed at reducing psychological distress among healthcare professionals [35]. These have led to gradual improvements in workforce resilience. By contrast, the strikingly high prevalence reported in this study suggests that such measures are either absent, insufficiently implemented, or inaccessible in this setting. These findings imply that health workers in the Kintampo North Municipal Hospital had high to low feelings of unexpected upset, inability to control situations, nervousness, inability to cope with situations, and anger regarding situations outside their control. Consequently, these are likely to influence job performance, quality of patient care, overall worker retention, productivity, and overall well-being.

In the Kintampo North Municipal Hospital, several factors may account for these elevated rates of psychological distress. Predominant among them are systemic challenges, including inadequate staffing, high patient-to-provider ratios, unusually high patient inflows, and limited resources, all of which intensify workload and emotional strain [21]. In addition, the absence of formal mental health support structures within healthcare institutions may intensify the risk, leaving workers with little opportunity to seek help. Also, sociocultural influences such as stigma associated with mental illness and cultural expectations of resilience among health workers may further discourage open acknowledgement or treatment of psychological symptoms [36]. Taken together, the prevalence of stress, depression, and anxiety reported in this study not only reinforces concerns about the mental well-being of healthcare workers in resource-constrained settings but also highlights the urgent need for institutional and policy-level interventions. Integrating mental health screening and support into routine occupational health programs, improving staffing and resource allocation, and implementing community-level strategies to reduce stigma could help safeguard the well-being of healthcare workers and ensure sustained quality of care delivery.

### Factors associated with stress, depression, and anxiety among health workers

This study also assessed a series of factors influencing stress, depression, and anxiety among the health workers studied. Among all the correlates, the most consistently significant and recurrent across all three conditions were job dissatisfaction, working in a shift rotational pattern, increased workload, chronic illnesses, uptake of alcohol, inadequate sleep duration, long working hours, male gender, and occupational role. Importantly, these risk factors are intensified by the specific challenges of working in a high-demand, resource-limited setting like Kintampo North.

In particular, most health workers reported dissatisfaction with their current jobs, leading to higher odds of experiencing stress, depression, and anxiety. This observation is consistent with previous reports that also found an association between job dissatisfaction and stress, depression, and anxiety [3,37]. Notably, this is likely to be a sentiment driven by several challenging working conditions within the hospital setting, including heavy workloads, limited availability of essential equipment and low salaries that do not adequately compensate for the demanding nature of their roles [3]. These issues, compounded by systemic challenges in the region, create a work environment that many health workers find unsustainable and unfulfilling. In disease- and accident-prone areas like the Kintampo North municipality, health workers often face high patient volumes and demanding workloads due to frequent health emergencies. Dissatisfaction could make it difficult for health workers to meet job demands effectively because they try to balance patient care with systemic limitations.

Secondly, working in a rotational shift pattern was highlighted by most health workers as a significant work-related factor contributing to the observed mental health disorders. This observation is consistent with a series of findings from previous studies [3,32]. The irregular and often unpredictable nature of shift work disrupts the body’s natural circadian rhythms, leading to chronic sleep disturbances, fatigue, reduced alertness, and impaired cognitive functioning [38]. This constant disruption negatively impacts both physical health and mental well-being due to a consistent lack of rest [39]. In Kintampo North, the frequent health emergencies and unpredictable patient inflow further compound the issue. As a result, healthcare workers are often required to remain alert, despite the strain caused by disrupted sleep patterns. Likewise, frequent work shifts disrupt personal lives and may isolate health workers from friends and family. This affects their support systems and can further impact their personal and professional well-being [40]

Furthermore, an increase in workload was revealed to be another work-related factor influencing stress, depression, and anxiety among health workers. This observation was also supported by earlier reports from a series of studies [3,41]. Health workers in such disease- and accident-prone areas often have to deal with time-sensitive situations that demand quick and accurate decisions. This is especially true in the context of emergency healthcare, where workers are under pressure to manage acute conditions, sometimes with limited resources. As the demands of roles escalate, they often find themselves overwhelmed by the sheer volume of tasks they must complete within limited timeframes [42]. This struggle to meet expectations leads to not only physical exhaustion but also psychological stress [43].

This study also revealed a significant association between chronic illness and various stressors, depression, and anxiety, which mirrors some reports from previous studies [3,44]. Chronic illnesses are likely to significantly contribute to mental health disorders by imposing ongoing physical, emotional, and financial burdens on individuals [45]. Physically, most chronic conditions require constant management, which can lead to physical discomfort [46]. Emotionally, living with a chronic illness can be attributed to feelings of helplessness, anxiety, and depression [47]. Affected individuals contend with lifestyle changes and thus limit activities that they previously enjoyed. Financially, the cost of ongoing treatments, medications, and potential loss of income due to reduced work capacity is likely to increase fatigue and increased vulnerability to additional health issues [45].

Moreover, the study found a significant association between alcohol uptake and the three levels of mental health disorders, which is consistent with earlier reports [48,49]. Alcohol consumption among healthcare workers, often sought as a coping mechanism for the stress, depression, and anxiety of their demanding roles, could paradoxically intensify the very mental health disorders they aimed to alleviate [50]. Many healthcare workers face prolonged hours, high-stakes decision-making, and emotional strain from patient outcomes, leading some to rely on alcohol as a means of temporary relief [51]. However, the effect of alcohol on the brain is likely to create a cycle of dependence that intensifies stress over time [52]. Physiologically, alcohol disrupts the balance of neurotransmitters, specifically affecting gamma-aminobutyric acid (GABA) and dopamine [53]. The disruption of these neurotransmitters could further lead to alteration of the hypothalamic-pituitary-adrenal (HPA) axis, which plays a crucial role in stress response [54]. These factors consequently create a detrimental feedback loop, where stress promotes drinking, and drinking intensifies stress [50].

In addition, the study revealed a significant association between long daily work duration and increased risk of stress, depression, and anxiety, which is also consistent with reports from previous studies [55,56]. This association could be attributed to physical exhaustion, mental fatigue, and emotional strain [45]. Long daily hours reduce recovery time, disrupt sleep, and impair focus, thereby increasing stress, depression, and anxiety [57]. A prolonged work duration, in turn, compounds these effects and consequently leaves limited time for personal activities and rest [58].

Moreover, less daily sleep duration was revealed as a work-related factor significantly associated with an increased likelihood of stress, depression, and anxiety among health workers, affirming a series of previous reports [59,60]. Less sleep duration has been found to disrupt the balance of stress hormones, especially cortisol [61]. With inadequate sleep, cortisol levels tend to remain elevated, and when combined with fatigue from lack of rest, the brain’s ability to manage emotions effectively is affected, which could potentially lead to increased irritability [61]. Furthermore, insufficient sleep impairs cognitive functions such as decision-making, problem-solving, and memory, making it harder for health workers to cope with daily challenges and increasing susceptibility to stress, depression, and anxiety [62]. Consequently, chronic sleep deprivation also weakens the immune system, making the body more vulnerable to illnesses and intensifying a series of mental health disorders [63].

Similarly, it was revealed that male healthcare workers had an increased likelihood of experiencing stress, depression, and anxiety, which mirrors earlier reports of stress [3,64]. This phenomenon could be attributed to a combination of cultural and systemic factors prevalent in Ghana. One possible explanation lies in the societal perceptions of masculinity, which often discourage emotional openness and the seeking of mental health support among men [65]. In many African cultures, including Ghana, masculinity is strongly associated with physical strength, resilience, and the ability to bear heavy burdens [65]. These cultural expectations may shape male healthcare workers’ reluctance to acknowledge or express emotional difficulties, including stress, depression, and anxiety. Furthermore, in healthcare settings in Ghana, men are frequently tasked with physically demanding roles or leadership positions, such as physicians, nurses in critical care units, and emergency medical personnel, all of which come with high levels of responsibility and stress [66].

These roles are often seen as a complement to their physical abilities and are marked by long hours, high patient loads, and inadequate resources. In such settings, the lack of mental health resources or support programs in many hospitals further exacerbates the situation. Male healthcare workers, in particular, may internalise their emotional distress due to the societal pressure to remain strong. This may delay the acknowledgement and treatment of their stress symptoms, leading to escalating mental health issues [67]. Moreover, this trend may be compounded by workplace norms and resource limitations in Ghana, where healthcare workers are often required to do more with less. Chronic understaffing, inadequate equipment, and high workloads are common challenges in many public hospitals in Ghana [68]. These systemic issues disproportionately affect male healthcare workers in physically taxing roles, making them more vulnerable to the detrimental effects of stress, depression, and anxiety [69,70]. Given that men may be more likely to suppress emotional expression and help-seeking behaviours, their mental health concerns may go unaddressed until they become more severe, thereby negatively impacting their well-being and job performance.

Finally, the study revealed a significant association between stress, depression, anxiety and occupational roles. Nurses, Allied Health Personnel, Physicians, Emergency Medical Technicians (EMTs), and Dispensary Technicians had an increased odds of experiencing all mental health disorders. This observation is in agreement with results from previous studies in different geographical settings [3,21,45]. The association could be due to the demanding nature of their roles. For instance, Nurses and Allied Health Personnel frequently encounter high patient loads, long shifts, and emotional situations that require continuous care under time pressures [45]. Similarly, Physicians may be faced with the additional responsibility of decision-making, the fear of diagnostic errors, and an overwhelming administrative burden [71]. Likewise, EMTs are always on alert for high-stakes emergencies and rapid response expectations. They are mostly exposed to trauma from emergency scenes, with little idle time for recovery [72]. Dispensary technicians, meanwhile, must manage meticulous tasks, such as accurate medication administration and patient consultations [73]. Together, these healthcare professionals face challenges that are mentally, physically, and emotionally demanding, creating an environment that could induce stress, depression, and anxiety.

### Discussion in relation to the Job Demands-Resources (JD-R) Model

The Job Demands-Resources (JD-R) Model offers a valuable lens for understanding how work-related factors influence the mental well-being of health workers in the Kintampo South Municipal Hospital. Our study highlights several job demands as significant factors associated with stress, depression, and anxiety among health workers. These findings align closely with the JD-R model, which posits that when job demands become overwhelming, they could lead to negative mental health outcomes [17]. Health workers who reported dissatisfaction with their jobs were significantly more likely to experience high levels of stress, depression, and anxiety. According to the JD-R model, job demands lead to health and motivational impairment. Thus, this manifests when the demands of the job outweigh the intrinsic satisfaction and sense of achievement derived from the work itself [74].

Similarly, increased workload was found to be another critical factor influencing mental health disorders among the health workers. The JD-R model asserts that an excessive workload is a primary job demand that leads to burnout [74], particularly when workers feel they cannot meet these demands. In Kintampo North Municipality, health workers are often overwhelmed by high patient volumes and frequent emergencies, which compounds their stress and increases the likelihood of experiencing anxiety and depression. Moreover, the negative impact of long working hours on mental health is well-documented in the literature, and our study confirms this trend. Evidently, the JD-R model also highlights that long work hours reduce time for recovery, leading to both physical and emotional strain [17]. In our study, health workers who were exposed to long or irregular shifts reported significantly higher levels of stress and anxiety.

In addition to job demands, sleep duration emerged as a significant factor influencing mental health outcomes. The JD-R model highlights the importance of personal resources, such as sleep, in helping workers recover from job demands [19]. Our study found that health workers who slept for fewer than six hours per day had significantly higher odds of experiencing stress, depression, and anxiety. Insufficient sleep not only impairs cognitive function but also increases emotional vulnerability, making it more difficult for workers to cope with the demands of their roles. According to the JD-R model, sleep could be considered a crucial personal resource that helps buffer the negative effects of high job demands [19,75]. However, when sleep is insufficient due to long working hours or irregular shifts, workers have fewer resources to manage stress, which increases the likelihood of developing mental health issues [75].

Our study found that male health workers had significantly higher odds of experiencing stress, depression, and anxiety compared to their female counterparts. This finding could be interpreted in light of the JD-R model’s assertion that gender roles and expectations may influence how workers perceive and cope with job demands [17]. Specifically, male workers may be less likely to seek help for mental health issues due to societal norms that discourage vulnerability, leading to greater internalization of stress and emotional strain. Furthermore, occupational role emerged as a significant predictor of mental health outcomes. Health workers in more demanding roles, such as nurses, allied health personnel, and physicians, were found to have significantly higher odds of experiencing stress, depression, and anxiety compared to orderlies. This observation aligns with the JD-R model’s assertion that employees in roles with higher job demands are more likely to experience burnout due to the intensity of their responsibilities [76]. Nurses and other healthcare professionals working in emergency settings or with high-stress patient cases are particularly vulnerable to emotional exhaustion and mental health distress, as they face continuous exposure to traumatic situations, critical care, and emotional strain.

### Policy implications

The findings of this study highlight the pressing need for public health, mental health, and occupational health and safety interventions to prioritise the mental well-being of healthcare workers, especially in resource-limited and high-demand settings like Kintampo North Municipality. Consequently, national and municipal health authorities should integrate mental health surveillance and support into routine public health planning. Furthermore, policies should mandate mental health risk assessments and resilience training as essential components of pandemic preparedness and health system strengthening efforts. In parallel, mental health should be embedded as a core component of workplace health programs. To this end, facilities must be adequately resourced to provide confidential and stigma-free access to mental health services such as counselling, stress management programs, and peer-support systems. Moreover, government-led initiatives should include targeted investments in mental health infrastructure within hospitals, alongside nationwide campaigns to destigmatise mental health challenges among healthcare professionals. Similarly, the occupational risks highlighted in this study necessitate stronger workplace protection. Accordingly, the Ghana Health Service and other relevant agencies should revise institutional policies to address shift patterns, promote work-hour regulation, and enforce limits on mandatory overtime. In addition, regular risk assessments and occupational health audits should be conducted. Finally, mechanisms for reporting psychosocial hazards and accessing occupational health support must be transparent, confidential and accessible to all health workers.

### Strengths and limitations

In an accident-prone and infectious disease hotspot like Kintampo North Municipality, this study contributes new insights into the mental health status of health workers. By employing a multifaceted strategy to assess both work-related and sociodemographic variables related to stress, depression, and anxiety, the study offers valuable recommendations to enhance the health and well-being of the workforce. However, several limitations must be acknowledged. To begin with, this study is cross-sectional in nature, providing only a snapshot of the mental health status of health workers at a specific point in time. As such, causal inferences cannot be made, and any potential changes in stress, depression, or anxiety over time cannot be tracked. This limitation also restricts the ability to assess the directionality of the observed associations. In addition, the reliance on self-reported data for measuring psychological outcomes introduces the possibility of response bias. Participants may underreport or overreport their levels of stress, depression, or anxiety due to social desirability or personal perceptions, which could skew the findings. Moreover, the study lacks triangulation with other data sources, such as qualitative insights or physiological indicators, which could provide a more comprehensive understanding of the mental health challenges faced by healthcare workers. Finally, the study was conducted at a single site within Kintampo North Municipality, limiting the external validity and generalizability of the findings. The results may not apply to healthcare workers in other regions or settings with different working conditions or healthcare infrastructures.

## Conclusion, recommendations, and future research direction

This study revealed a high prevalence of stress (66.5%), depression (63.6%), and anxiety (87.9%) among health workers in Kintampo North Municipal Hospital, with determinants spanning occupational, lifestyle, and demographic domains. Factors such as job dissatisfaction, rotational shifts, long working hours, insufficient sleep, chronic illness, alcohol use, male gender, and occupational role significantly increase the risk of mental health disorders. The prevalence and correlates of these disorders highlight the need for evidence-based policies to reduce these mental health disorders and their associated complications. These include improving work-life balance, flexible work schedules, reducing mandatory overtime, and introducing regular but scheduled breaks. In the same vein, job dissatisfaction needs to be addressed through sufficient staffing and adequate tools and equipment. Programs that regularly recognise staff for their work and allow for mental health days could also give a sense of value and reduce occupational stress. Moreover, settings should integrate on-site mental health support, such as access to counselling services, stress management training, and peer-support groups in healthcare settings. Health workers should be encouraged to use these resources free of stigma. Finally, future studies should focus on investigating the long-term effects of occupational stress on the health of healthcare workers so that more targeted interventions can be developed to mitigate the health effects of chronic stress. Likewise, experimental studies evaluating the effectiveness of workplace policies such as revised shift schedules, increased staffing, and access to mental health services might provide evidence for scalable interventions that enhance worker satisfaction and decrease stress.

## Supporting information

S1 Text Questionnaire. (DOCX)

S1 Data Dataset. (XLSX)
