# Supplementary material for: Mental health burden among healthcare workers in Kintampo North Municipal Hospital: A descriptive analysis of stress, depression, and anxiety based on Job Demands-Resources (JD-R) model
Source: PLOS Ment Health. 2025 Dec 3;2(12):e0000478. doi: 10.1371/journal.pmen.0000478 (PMC12798270; doi:10.1371/journal.pmen.0000478)
Supplement: S1 Text — (DOCX) [file pmen.0000478.s001.docx]

**QUESTIONNAIRE**

Akenten Appiah Menka University of Skill Training and Entrepreneurial Development

Department of Public Health Education

Survey on occupational health and safety

This questionnaire is part of a survey for MPhil work in Occupational Safety and Health Education being undertaken by Mohammed Zakaria a student at the Department of Public Health Education, Akenten Appiah Menka University of Skill Training and Entrepreneurial Development. The objective of this research is to assess the mental health and well-being of health workers in Kintampo North Municipality and to identify the associated implications for occupational health and safety within the health service. The study is based on a selected sample, so your participation is critical. I would therefore be most grateful if you could spare a few minutes of your time to complete this questionnaire. You are assured that any information you provide will be treated with strict confidentiality. Your anonymity is also guaranteed. Your participation is voluntary.

**Instruction: Please tick [✓] where appropriate, and for others, you may specify by writing.**

Name of interviewer:

Signature:

Date:

Time interview started:

# SECTION A: SOCIODEMOGRAPHIC CHARACTERISTICS OF PERSONNEL

| No. | Question | Response options | Skip |
| --- | --- | --- | --- |
|  | Gender | 1. Male 2. Female |  |
|  | Age |  |  |
|  | Age Category | 1. Between 18 and 25 2. Between 26 and 30 3. Between 31 and 40 4. Between 41 and 50 5. Between 51 and 60 |  |
|  | Marital Status: | 1. Never married 2. Married 3. Cohabitation 4. Separated 5. Divorced 6. Widowed |  |
|  | Educational Level | 1. SHS/Secondary/Technical 2. Certificate 3. Diploma 4. Degree |  |
|  | Religious Affiliation | 1. Islam 2. Christianity 3. African Traditional Religion 4. Others |  |
|  | Occupational status | 1. Nurses 2. Allied Health Personnel 3. Physicians 4. Emergency Medical Technicians 5. Dispensary Technicians 6. Orderlies |  |
|  | Number of years in the health profession | 1. Between 1 and 7 2. Between 8 and 12 3. Between 13 and 17 4. Between 18 and 23 |  |
|  | Department | 1. Administration 2. Out-Patient Department 3. Ward 4. Mortuary 5. Dispatch/control 6. Operations |  |
|  | On the average, how much do you earn from your work? | 1. Less than 1500 cedis 2. Between 1500 – 2000 cedis 3. Between 2100 – 2900 cedis 4. Between 3000 cedis and above |  |

# SECTION B: PREVALENCE OF STRESS, BURNOUT, DEPRESSION, AND ANXIETY AMONG HEALTH WORKERS. SELECT BY TICKING (√) THE APPROPRIATE OPTIONS

| No. | Question | Response options | Skip |
| --- | --- | --- | --- |
| **PREVALENCE OF STRESS**  **BECK’S PERCEIVED STRESS INVENTORY**  Please read each statement and tick (√) a number 1,2, 3 or 4 which indicates how much the statement applied to you after experiencing the triggers over the past month. There are no right or wrong answers.  **In the last month, how often have you been/felt:** | | | |
|  | Upset because of something that happened unexpectedly? | 1. Never 2. Sometimes 3. Often 4. Almost always |  |
|  | You were unable to control the important things in your life? | 1. Never 2. Sometimes 3. Often 4. Almost always |  |
|  | Nervous and stressed? | 1. Never 2. Sometimes 3. Often 4. Almost always |  |
|  | You could not cope with all the things that you had to do? | 1. Never 2. Sometimes 3. Often 4. Almost always |  |
|  | Angered because of things that happened that were outside of your control? | 1. Never 2. Sometimes 3. Often 4. Almost always |  |
|  | Difficulties were piling up so high that you could not overcome them? | 1. Never 2. Sometimes 3. Often 4. Almost always |  |
|  | Confident about your ability to handle your personal problems? | 1. Never 2. Sometimes 3. Often 4. Almost always |  |
|  | Things were going your way? | 1. Never 2. Sometimes 3. Often 4. Almost always |  |
|  | Able to control irritations in your life? | 1. Never 2. Sometimes 3. Often 4. Almost always |  |
|  | You were on top of things? | 1. Never 2. Sometimes 3. Often 4. Almost always |  |

# SECTION C: COPING MECHANISMS. ANSWER THE QUESTIONS BELOW BY TICKING THE APPROPRIATE OPTION WHERE APPLICABLE.

| Please read each statement and tick (√) a number 1,2, 3 or 4 which indicates how much the statement applied to you after experiencing the triggers over the past month. There are no right or wrong answers. | | | |
| --- | --- | --- | --- |
| No. | Question | Response options | Skip |
| **Stress Management:** | | | |
|  | Practicing Mindfulness or Meditation | 1. Never 2. Sometimes 3. Often 4. Almost always |  |
|  | Engaging in Physical Exercise | 1. Never 2. Sometimes 3. Often 4. Almost always |  |
|  | Seeking Support from Colleagues | 1. Never 2. Sometimes 3. Often 4. Almost always |  |
|  | Seeking Support from Specialists | 1. Never 2. Sometimes 3. Often 4. Almost always |  |
|  | Setting Realistic Work Goals | 1. Never 2. Sometimes 3. Often 4. Almost always |  |
|  | Taking Short Breaks During Work Hours | 1. Never 2. Sometimes 3. Often 4. Almost always |  |
|  | Connecting with your community- or faith-based organizations. | 1. Never 2. Sometimes 3. Often 4. Almost always |  |
|  | Lowering your expectations. | 1. Never 2. Sometimes 3. Often 4. Almost always |  |
|  | Maintaining emotionally supportive relationships. | 1. Never 2. Sometimes 3. Often 4. Almost always |  |
|  | Maintaining emotional composure or expressing distressing emotions. | 1. Never 2. Sometimes 3. Often 4. Almost always |  |
|  | Challenging previously held beliefs that are no longer adaptive | 1. Never 2. Sometimes 3. Often 4. Almost always |  |
|  | Directly attempting to change the source of stress | 1. Never 2. Sometimes 3. Often 4. Almost always |  |
|  | Distancing yourself from the source of stress | 1. Never 2. Sometimes 3. Often 4. Almost always |  |
|  | Eating well | 1. Never 2. Sometimes 3. Often 4. Almost always |  |
|  | Limiting alcohol and stimulants | 1. Never 2. Sometimes 3. Often 4. Almost always |  |
| **General Well-being:** | | | |
|  | Is mental health support provided by your workplace? | 1. Yes 2. No |  |
|  | If yes, how often | 1. Daily 2. Weekly 3. Monthly 4. Quarterly 5. Biannually 6. Annually 7. No definite time-fixed |  |
|  | Overall, how satisfied are you with the mental health support provided by your workplace? | 1. Very Dissatisfied 2. Dissatisfied 3. Neutral 4. Satisfied 5. Very Satisfied |  |
|  | Do you meet as personnel to discuss mental and safety issues? | 1. Yes 2. No |  |
|  | If yes, how often | 1. Daily 2. Weekly 3. Monthly 4. Quarterly 5. Biannually 6. Annually 7. No definite time-fixed |  |
|  | How likely are you to recommend the coping mechanisms you find effective to your colleagues? | 1. Very unlikely 2. Unlikely 3. Neutral 4. Likely 5. Very likely |  |
|  | Are there any additional coping mechanisms or support systems you find effective that are not listed above? (Open-ended) | 1. Yes  2. No |  |
|  | If Yes, specify |  |  |
